# A Specific A/T Polymorphism in Western Tyrosine Phosphorylation B-Motifs Regulates *Helicobacter pylori* CagA Epithelial Cell Interactions

**DOI:** 10.1371/journal.ppat.1004621

**Published:** 2015-02-03

**Authors:** Xue-Song Zhang, Nicole Tegtmeyer, Leah Traube, Shawn Jindal, Guillermo Perez-Perez, Heinrich Sticht, Steffen Backert, Martin J. Blaser

**Affiliations:** 1 Departments of Medicine and Microbiology, New York University School of Medicine and VA Medical Center, New York, New York, United States of America; 2 Friedrich Alexander University Erlangen, Department of Biology, Division of Microbiology, Erlangen, Germany; 3 Friedrich Alexander University Erlangen, Bioinformatics, Institute for Biochemistry, Erlangen, Germany; University of Illinois, UNITED STATES

## Abstract

*Helicobacter pylori* persistently colonizes the human stomach, with mixed roles in human health. The CagA protein, a key host-interaction factor, is translocated by a type IV secretion system into host epithelial cells, where its EPIYA tyrosine phosphorylation motifs (TPMs) are recognized by host cell kinases, leading to multiple host cell signaling cascades. The CagA TPMs have been described as type A, B, C or D, each with a specific conserved amino acid sequence surrounding EPIYA. Database searching revealed strong non-random distribution of the B-motifs (including EPIYA and EPIYT) in Western *H. pylori* isolates. *In silico* analysis of Western *H. pylori* CagA sequences provided evidence that the EPIYT B-TPMs are significantly less associated with gastric cancer than the EPIYA B-TPMs. By generating and using a phosphorylated CagA B-TPM-specific antibody, we demonstrated the phosphorylated state of the CagA B-TPM EPIYT during *H. pylori* co-culture with host cells. We also showed that within host cells, CagA interaction with phosphoinositol 3-kinase (PI3-kinase) was B-TPM tyrosine-phosphorylation-dependent, and the recombinant CagA with EPIYT B-TPM had higher affinity to PI3-kinase and enhanced induction of AKT than the isogenic CagA with EPIYA B-TPM. Structural modeling of the CagA B-TPM motif bound to PI3-kinase indicated that the threonine residue at the pY+1 position forms a side-chain hydrogen bond to N-417 of PI3-kinase, which cannot be formed by alanine. During co-culture with AGS cells, an *H. pylori* strain with a CagA EPIYT B-TPM had significantly attenuated induction of interleukin-8 and hummingbird phenotype, compared to the isogenic strain with B-TPM EPIYA. These results suggest that the A/T polymorphisms could regulate CagA activity through interfering with host signaling pathways related to carcinogenesis, thus influencing cancer risk.

## Author Summary

As the dominant bacterium living in the human stomach, *Helicobacter pylori* has mixed roles in host health. One significant pathogenic risk factor is the CagA protein, which interferes with multiple host cell signaling pathways through its EPIYA tyrosine phosphorylation motifs (TPMs). Through database searching and silico analysis, we reveal a strong non-random distribution of the EPIYA B motif polymorphisms (including EPIYT and EPIYA) in Western *H. pylori* isolates, and provide evidence that the EPIYT are significantly less associated with gastric cancer than the EPIYA. By constructing a series of *H. pylori cagA* isogenic mutants and isogenic complementation plasmids, generating specific antibodies, co-culturing with human AGS cells, performing biochemical and modeling analysis, we demonstrate that CagA B-motif phosphorylation status is essential for its interaction with host PI3-kinase during colonization and that CagA with an EPIYT B-motif had significantly attenuated induction of interleukin-8 and the hummingbird phenotype, had higher affinity with PI3-kinase, and enhanced induction of AKT compared to the EPIYA. These findings provide insight into how Western *H. pylori* CagA regulates cancer-related activity inside host cells through the A/T polymorphisms at the functionally important B motif.

## Introduction

*Helicobacter pylori*, a spiral-shaped, microaerophilic gram-negative bacterium, persistently colonizes the human gastric mucosa [1,2]. *H. pylori* is carried by about 50% of the world’s population, and it exhibits extensive genetic diversity and distinct phylogeographic features [3,4]. Colonization increases risk of peptic ulcer disease and gastric carcinoma [5,6], and has been associated with diminished risk for esophageal inflammatory and neoplastic lesions [7,8], and childhood-onset asthma [9,10]. In 1995, the cytotoxin-associated gene A (CagA) protein of *H. pylori* was first associated with increased risk of gastric cancer [11], and since then, its pathogenic effects have been intensely studied [1,12].

The 120–145 kDa CagA protein is encoded by the *cagA* gene, located within the ∼40 kb *H. pylori cag* pathogenicity island (*cag*PAI) [13,14], along with a type IV secretion system that injects it into host gastric epithelial cells [15]. The carboxy-terminal region of CagA has several Glu-Pro-Ile-Tyr-Ala (EPIYA) motifs which are strongly correlated to gastric disease outcomes [16,17]. The carboxy-terminal region of CagAs exhibit geographical, structural, and functional diversity, which is the result of the evolution of this protein through various modes of recombination mechanism [18].

In host cells, CagA molecules are associated with the inner surface of the plasma membrane and are dimerized via the carboxy-terminal EPIYA motif-containing regions [19,20]. CagA molecules undergo tyrosine phosphorylation (pCagA) at the EPIYA motifs by host Src-family kinases (SFKs) and Abl kinase [21–23]. CagA interacts with multiple host signaling factors through its EPIYA TPMs in a phosphorylation-dependent or -independent manner [24,25], affecting cell proliferation, motility, polarity, apoptosis, inflammation and nuclear responses, which may promote gastric carcinogenesis [26–28]. By mimicking host substrates through its C-terminal sequence, CagA inhibits PAR1/MARK family kinase pathways [29], and by association with the human tumor suppressor apoptosis-stimulating protein of p53-2 (ASPP2) through its N-terminal sequence, CagA inhibits apoptosis of host cells co-colonized with *H. pylori* [30]. Through phosphorylated EPIYA TPMs, pCagA binds to the Src homology 2 (SH2) domains of host signaling factors [26,28]. In this way pCagA activates the tyrosine phosphatase Shp2, which affects cell proliferation by inducing the ERK MAP kinase cascade [31–33], and also leads to cell elongation (producing the hummingbird phenotype) by inhibition of focal adhesion kinase (FAK) [34–36]. Phosphorylated TPMs also facilitate CagA interactions with C-terminal Src kinase (CSK), which inhibits SFK activity and negatively regulates CagA-Shp2 interaction [37]. The phosphorylated CagA TPMs directly bind other tyrosine phosphatases such as Shp1, phosphatidylinositide 3-kinase (PI3-kinase) and GTPase activating protein Ras GAP1, as well as adaptor proteins Crk-I, Crk-II, Crk-L, Grb2, and Grb7 [12,26]. Transgenic mice expressing wild-type CagA but not tyrosine-phosphorylation-resistant CagA developed gastric and small intestinal epithelial hyperplasia and neoplasia and B cell lymphomas and myeloid leukemias [38], supporting a critical role of CagA tyrosine phosphorylation in *H. pylori*-induced oncogenesis.

In addition to phosphorylation-dependent effects, CagA also associates with the polarity-regulating kinase partitioning-defective 1 (PAR1) protein through its C-terminal CagA-multimerization motif (CM), which overlaps with the EPIYA C- or D-TPM sequences [33,39]. The interaction between CagA and Par1 disrupts gastric epithelial cell tight junctions and apical-basal polarity [33], and enhances CagA TPM-phosphorylation-dependent interactions by stabilizing complex structures such as CagA-Shp2 [20]. In total, both phosphorylation-dependent and -independent [33–35] interactions affect host signaling pathways.

*H. pylori* has extensive genetic diversity [40–42]; isolates from different populations exhibit distinct biogeographic features, reflecting ancient human migrations [43]; *cagA* also possesses population-specific polymorphisms with major East Asian and Western groupings [44,45]. Four distinct CagA EPIYA TPMs (A, B, C or D), have conserved flanking sequences [46]. East Asian CagA include A-, B-, and D-TPMs, while Western CagA has A-, B-, and C-TPMs [47]. The East Asian CagAs are more interactive with host cells than Western CagAs, largely due to the higher affinity of the strongly phosphorylated D-TPM to Shp-2 than the Western C-TPMs [47,48]. Western CagA includes one or multiple C-TPMs, while East Asian CagA only has one D-TPM [12].

Regardless of C/D type, most CagA molecules include single A- and B-TPMs that undergo later and not simultaneous tyrosine phosphorylation [25]. The phosphorylated A- or B-TPMs have distinct host interaction partners from C- or D-TPMs and from each other [26], suggesting unique signaling functions. Here we report and characterize the functional importance of a specific A/T polymorphism present only within the Western CagA EPIYA B-TPMs.

## Results

### Polymorphisms in the CagA tyrosine phosphorylation EPIYA motif

Based on the CagA sequences published in Genbank, we investigated the variability within the C-terminal EPIYAs. A total of 2,561 complete or partial *H. pylori* CagA protein sequences were analyzed for polymorphisms within the EPIYAs. Our analysis indicated that the CagA B-TPM exhibits the highest variability (Table 1). EPIYA represents only 72.6% of 2,617 B-TPM sequences, with 23 alternative sequences present, including EPIYT, ESIYT, ESIYA and GSIYD; EPIYT is the most frequent alternative. In contrast, very few alternative sequences are identified in the A- and C-TPMs (Table 1), and none in the 1,196 type D-TPMs; only a single alternative sequence (EPIYV) is observed in the 1,620 C-TPMs (Table 1). All 25 independent CagA sequences with the EPIYV C-TPM show the same rare B-TPM (GSIYD). Only one low frequency (0.2%) alternative sequence (EPIYT) is observed in the A-TPMs (Table 1). In total, these results indicate specific polymorphisms in the CagA tyrosine phosphorylation (EPIYA) sequences, especially involving the B-TPMs. Among the 35 fully-sequenced *H. pylori* genomes and 81 ongoing partially-sequenced *H. pylori* genomes possessing the *cag*PAI (based on the NCBI Bioproject Database), EPIYA represents 52.6%, while EPIYT represents 34.5% of the B-TPMs.

**Table 1:** Distribution of alternative sequences in the four types of *H. pylori* CagA TPMs^a^.

	**Number**					
**TPM type**	**Total**	**EPIYA**	**EPIYT**	**ESIYA**	**ESIYT**	**Other**
	**(n = 7954)**	**(n = 7207) (%)**	**(n = 478)**	**(n = 96)**	**(n = 60)**	**(n = 113)**
A	2521	2517 (99.8)	4	0	0	0
**B^b^**	**2617**	**1899 (72.6)**	**474**	**96**	**60**	**88^c^**
C	1620	1595 (98.5)	0	0	0	25^d^
D	1196	1196 (100)	0	0	0	0

^a^ From review of Genbank entries to Aug. 8, 2013.

^b^B-TPM bolded, since most variable.

^c^ B-domain other TPMs include: GSIYD (31), EPVYA (13), ELIYA (12), ESIYD (7), ETIYA (3), EPIYD (3), EHIYA (2), EPTYA (2), EPIYS (2), EPIYV (2), ESVYA (2), DPIYA (1), DPIYD (1), EPVYT (1), QPIYP (1), EPIFT (1), EPIHA (1), EPINA (1), ESIYS (1), EFIYT (1).

^d^ The C-domain other TPMs all are EPIYV (25), which only is present when the B-domain sequence is GSIYD (p<0.0001).

### The EPIYA- B-TPM of Western type CagAs has alternative sequences

Next, analyzing the B-TPMs in East Asian CagA possessing D-TPMs or Western type CagA possessing C-TPMs, we found that the distributions of the identified alternative sequences were significantly different (Table 2). In the Western CagA EPIYA B-TPMs, there are two major sequences (EPIYA; 55.5% and EPIYT; 32.9%). Other alternative TPMs comprise about 11.6% of the sequences. In contrast, among East Asian CagA B-TPMs, EPIYA is the major sequence (91.1%), EPIYT is at low (1.7%) frequency, and ESIYA is the alternative TPM with the highest frequency (6.0%) (Table 2). In total, Western CagAs have more alternative B-TPM EPIYA sequences than do East Asian CagAs.

**Table 2:** Distribution of alternative TPMs in 2617 CagA B-TPMs, by *H. pylori* geographic origin.

**B-TPM**	**Number (% of total)^a^**			
**Sequence type**	**Total**	**East Asian**		**Western**		**p-value^b^**
	**(n = 2617)**	**(n = 1190)**		**(n = 1337)**		
EPIYA	1899	1084	(91.1)	742	(55.5)	<0.001
EPIYT	474	20	(1.7)	440	(32.9)	<0.001
ESIYA	96	71	(6.0)	26	(1.9)	<0.001
ESIYT	60	7	(0.6)	49	(3.7)	<0.001
Others^C^	88	8	(0.7)	80	(6.0)	<0.001

^a^ For 93 Genbank records, strain origin was not recorded.

^b^ By Chi-square test.

^c^ See Table 1.

Since Western CagAs may have more than one C-TPM, we asked whether the presence of the alternative EPIYA B-TPMs is related to C-TPM number. There was no link between the alternative B-TPM sequences and the number of C-TPMs, except for ESIYT, which co-appears with 2 C-TPMs on the same Western CagAs at significantly high frequency (Table 3). These findings indicate a common previously unrecognized TPM polymorphism with strongly non-random distribution in the available census of strains.

**Table 3:** Relation of B-domain TPM sequence type to number of C-domain TPMs in 1322 Western CagA sequences.

**B-TPM Sequence type**	**Total^a^**	**C-TPM number (%)**			
	**(n = 1322)**	**1 (n = 1035)**	**2 (n = 254)**	**3 (n = 33)**	**p-value**
EPIYA	730	582(79.7)	124(17.0)	24(3.3)	NS^b^
EPIYT	436	339(77.8)	90(20.6)	7 (1.6)	NS
ESIYA	26	21 (80.8)	4(15.4)	1 (3.8)	NS
ESIYT	50	14 (28.0)	35(70.0)	1 (2.0)	<0.001
Others	80	79 (98.8)	1 (1.2)	0 (0.0)	NS

^a^ For some Western strains (n = 16), there are > 3 C-TPMs.

^b^ NS, P>0.05.

### Pathological association with the Western B-domain TPM alternatives

To assess the relationship of B-TPM sequence and clinical outcome, we analyzed a total of 364 Western CagAs, which were reported to be present in patients with defined gastrointestinal pathology, according to the descriptions from Genbank and the indicated publications (Table 4). Compared with gastritis alone, gastric cancer was significantly associated with the EPIYA B-TPMs, whereas duodenal ulcers were significantly associated with the EPIYT B-TPM (Table 4). That these polymorphisms in B-TPM are associated with different diseases suggest that EPIYT and EPIYA may differentially regulate the CagA pathophysiologic roles in Western *H. pylori* strains that interact with host cells; while the CM and CRPIA motifs are commonly present in both forms of CagA.

**Table 4:** Relation of Western CagA B-TPM sequence type to gastrointestinal ailments.

**Clinical Status^a^**	**Number (% of total)**			
	**Total**	**EPIYA**		**EPIYT**		**p-value^b^**
	**(n = 364)**	**(n = 194) (53.3%)**		**(n = 170) (46.7)**		
Gastritis alone	171	88	(51.5)	83	(48.5)	(Reference)
Duodenal Ulcer	102	43	(42.2)	59	(57.8)	0.02
Gastric Ulcer	24	13	(54.2)	11	(45.8)	NS
Gastric Cancer	46	35	(76.1)	11	(23.9)	0.0019
Esophagitis	21	15	(71.4)	6	(28.6)	NS

^a^Based on the descriptions from the Genbank database or the associated publications.

^b^ By X^2^ analysis; NS, P>0.05.

### Codon usage in the Western B-domain TPMs

**Figure 1:**
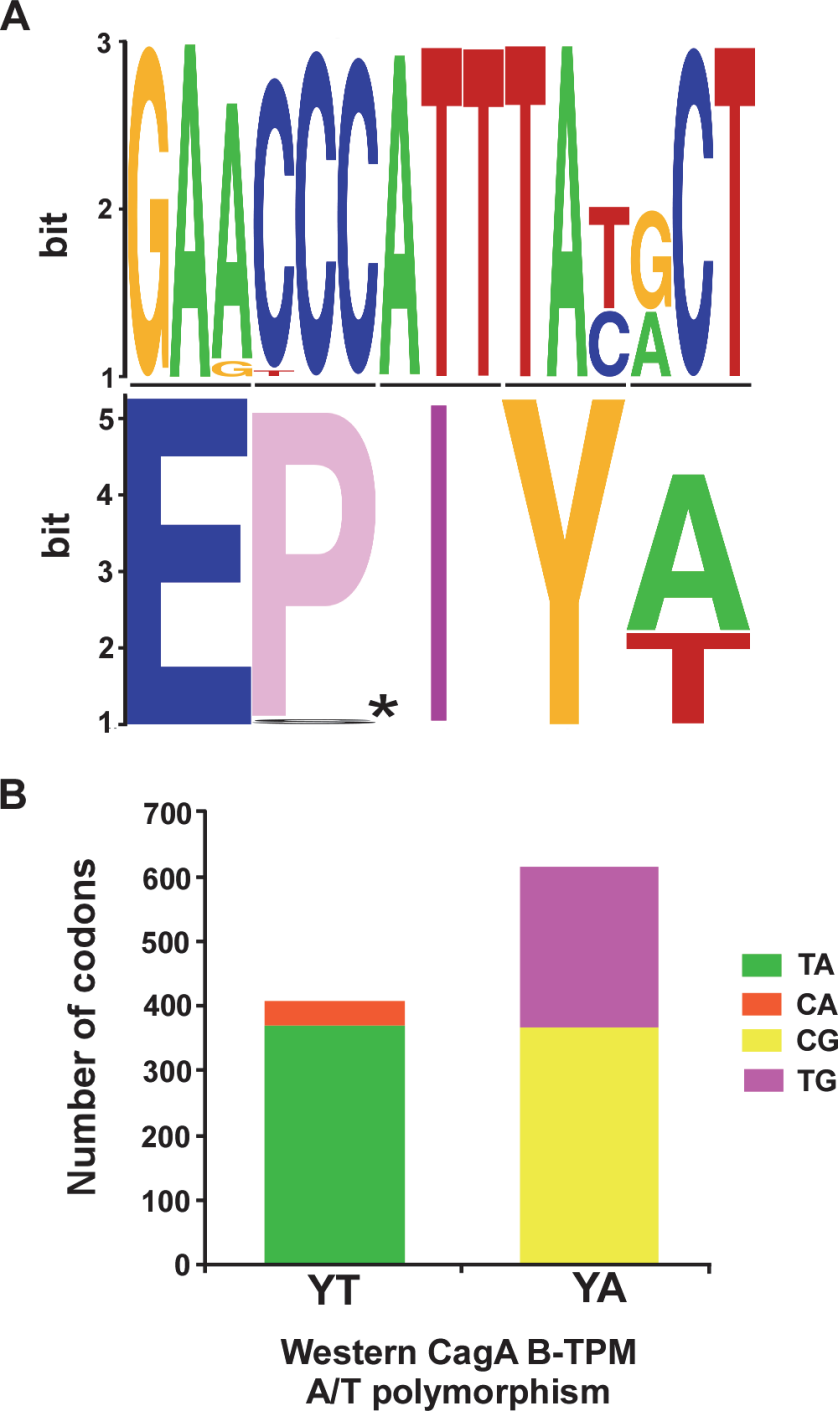
Polymorphisms of the Western CagA B-TPMs and their codons. **Panel A**: The sequences for 1027 Western CagA B-TPMs were aligned and a sequence logo was produced (*upper*), and amino acids (*lower*, *: E-P/S-I-). **Panel B**: The distribution of codons for the Western CagA B-TPM YA and YT polymorphisms. The proportions of major (TA) and minor (CA) codons of YT are significantly different (p<0.0001). The proportions of major (CG) and minor (CA) codons of YA and also are significantly different (p = 0.0075).

To assess whether the EPIYA/T polymorphisms at the protein level were random, we compared the codons in which the A/T polymorphisms were present (S1 Table in S1 Text and Fig. 1). Only one major (91.1%) set of codons (TAT ACT) encodes the YT of EPIYT B-TPM. In contrast, there are two major sets of codons (TAC GCT and TAT GCT) that encode the YA of the EPIYA B-TPMs with similar frequencies (59.4% and 40.4%, respectively). Compared with YA and YT codons in the other loci in the *H. pylori* 26695 genome, this distribution is significantly non-random (p<0.001). This non-random distribution suggests that the polymorphisms have been selected rather than being stochastic, potentially providing for different CagA functional roles.

### Construction of *H. pylori* isogenic *cagA* mutants with TPM polymorphisms

To investigate how the major (EPIYT) alternative TPM affects CagA functions, we created a series of isogenic *H. pylori* mutants that express a Western CagA with variant TPMs. The Western-type *cagA* gene from *H. pylori* strain 147C, originally isolated from a human antrum corpus [44], was used as the CagA parent gene for the constructions, created in a site-directed manner using a recombinant PCR technique (S1A Fig. in S1 Text). This 147C *cagA* gene possesses one A-, B- and C-TPM. The gene product CagA_147C_ has been previously shown to induce both AGS cell hummingbird phenotype and IL-8 production [44,49]. We replaced the *H. pylori* 26695 *cagA* ORF with isogenic *cagA* genes in its native genetic locus via transformation. The set of 6 isogenic *H. pylori* 26695 mutants all possess *cagA* with EPIYA, EPIYT, or EPIAT (as a control: presumed to be inactive) B-TPMs, and with EPIYA or EPIAA forms of the A- or C-TPM (S1A Fig. in S1 Text). These mutants exhibit the same physiological characters, transformation frequency and growth rates *in vitro* as the parental 26695 strains. Western blotting confirmed that each isogenic mutant expressed a CagA molecule (S1B Fig. in S1 Text), and sequencing of each *cagA* ORF confirmed that the sequences surrounding the target site(s) were identical.

### CagA EPIYT B-TPM is phosphorylated during *H. pylori* co-culture

**Figure 2:**
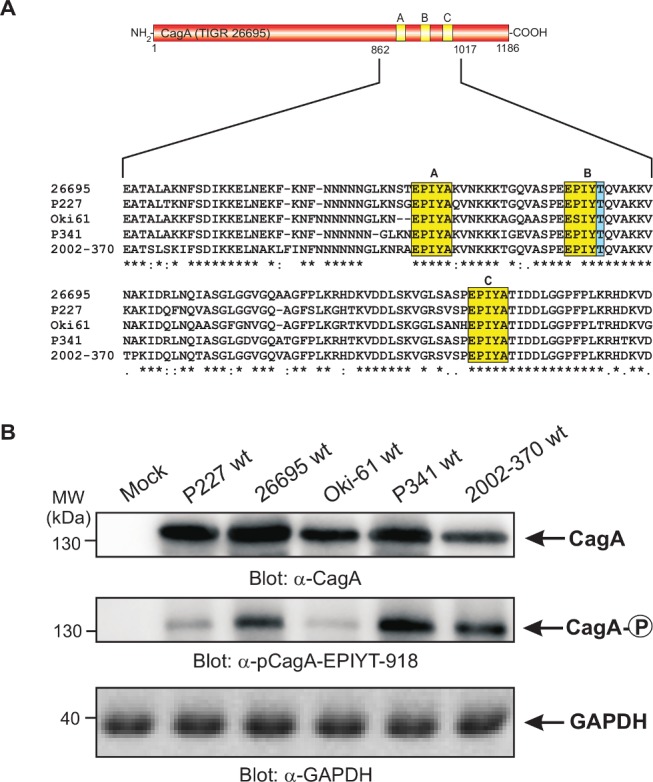
Sequence comparison of the three TPM sites in CagA proteins from different clinical *H. pylori* strains and specific detection of phosphorylated EPIYT-motif during co-culture. **Panel A**: CagA proteins of *H. pylori* vary in their carboxy-terminal TPM sites. These EPIYA-repeats serve as tyrosine phosphorylation sites of CagA and can be targeted by c-Abl and c-Src kinases. Three EPIYA- or EPIYT-segments at position A, B and C are shaded with yellow. One striking feature of B-TPM is the presence of a threonine residue in the +1 position (shaded with blue) relative to the phosphorylated tyrosine residue, which is highly conserved in most but not all *H. pylori* strains and may affect the capabilities of binding the p85 subunit of PI3-kinase, as discussed in the text. The CagA protein sequences were obtained from databases and sequence alignment was done using the ClustalW2 program (http://www.ebi.ac.uk/Tools/msa/clustalw2/). **Panel B**: To investigate whether the EPIYT-motif can be phosphorylated during co-culture, AGS cells were co-incubated with the indicated CagA-expressing *H. pylori* strains for 6 h. Phosphorylation of CagA was examined using the phospho-specific α-pCagA-EPIYT-918 antibody. Loading of equal amounts of protein in each sample was confirmed by probing with monoclonal α-CagA and α-GAPDH antibodies.

To investigate CagA B-TPM phosphorylation status and function during co-culture, we first generated phospho-specific and non-phospho antibodies, α-pCagA-EPIYT-918 (phospho) and α-CagA-EPIYT-918 (non-phospho) against the EPIYT B-TPM motif of CagA, corresponding to the amino acid residues derived from strain 26695 (S2 Fig. in S1 Text). Our analysis indicated that the α-pCagA-EPIYT-918 (phospho) or α-CagA-EPIYT-918 (non-phospho) antibodies recognize the B-TPM including both EPIYT B-TPM and EPIYA B-TPM, but not the A-TPM or C-TPM (control) peptides (S2 Fig. in S1 Text). To investigate whether this CagA EPIYT B-TPM can be phosphorylated during co-culture, AGS cells were co-incubated for 6 h with a set of clinical *H. pylori* strains, which vary in their CagA carboxy-terminal TPM sites. Phosphorylation of CagA at B-TPM was examined using the now-confirmed phospho-specific α-pCagA-EPIYT-918 antibody. Results indicated that the EPIYT-motif can be phosphorylated during co-culture, but to varying extents (Fig. 2).

### The alternative B-TPM EPIYT has enhanced PI3-kinase/AKT pathway induction

**Figure 3:**
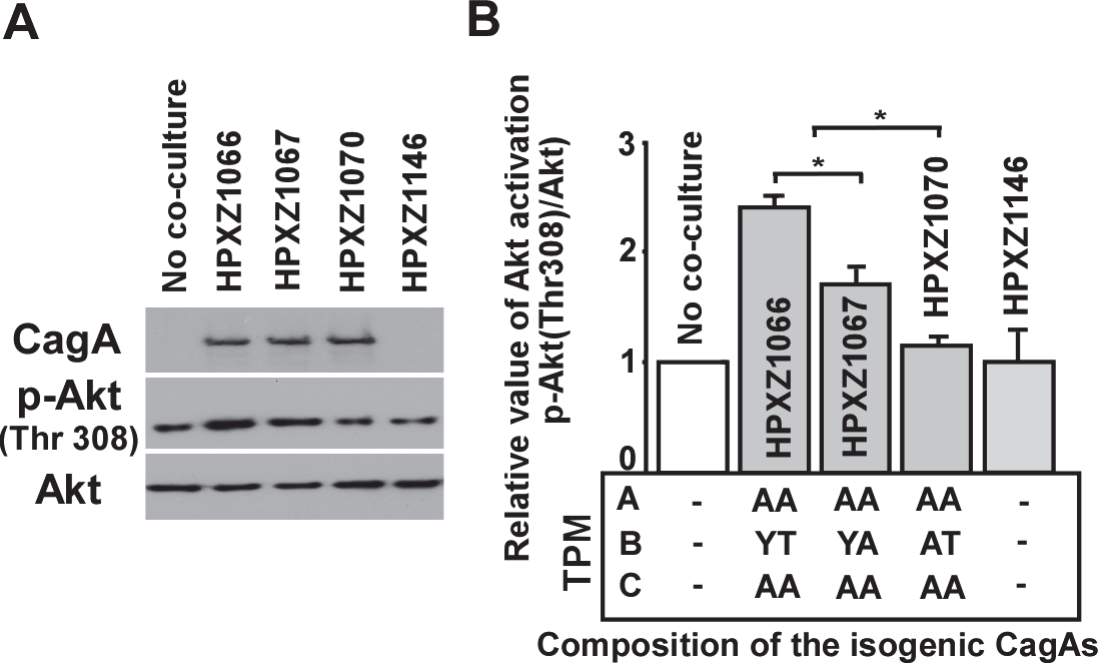
Analysis of the PI3-kinase-AKT pathway after co-culture of human AGS cells with isogenic *H. pylori* strains containing the engineered CagA molecules. **Panel A**: After 24 h co-culture with *H. pylori* isogenic *cagA* mutants, AGS cells were washed 5 times with ice-cold PBS buffer to remove *H. pylori* cells, and whole cell lysates were separated by SDS-PAGE, followed by immunodetection with antibodies (α-CagA, α-p-AKT, or α-AKT). **Panel B**: Targeted bands were quantified with ImageJ software to calculate the relative level of AKT phosphorylation.

To investigate how this alternative B-TPM affects the role of CagA in host signaling pathways, we co-cultured the isogenic *H. pylori* mutants with AGS cells for 24 h and analyzed cell lysates for CagA-mediated protein binding and signal activation by immunoblotting and co-immunoprecipitation. Co-culture with the EPIYT isogenic strain induced the phosphorylation of serine/threonine kinase AKT (also called protein kinase B, PKB) at threonine residue 308(T-308) 2.4 ± 0.10 fold, compared with 1.7 ± 0.14 fold for the EPIYA strain (Fig. 3), indicating intensified induction of PI3-kinase/AKT activation. Neither co-culture with the isogenic *H. pylori* strain possessing a B-domain with an abolished tyrosine phosphorylation site, nor co-culture with the *cagA* knockout strain significantly increased AKT phosphorylation at T-308 (Fig. 3), suggesting that CagA-positive *H. pylori* can activate the PI3-kinase/AKT pathway with activity dependent on B-domain TPM phosphorylation.

### The EPIYT site at B-TPM of CagA is necessary for interaction with PI3-kinase

**Figure 4:**
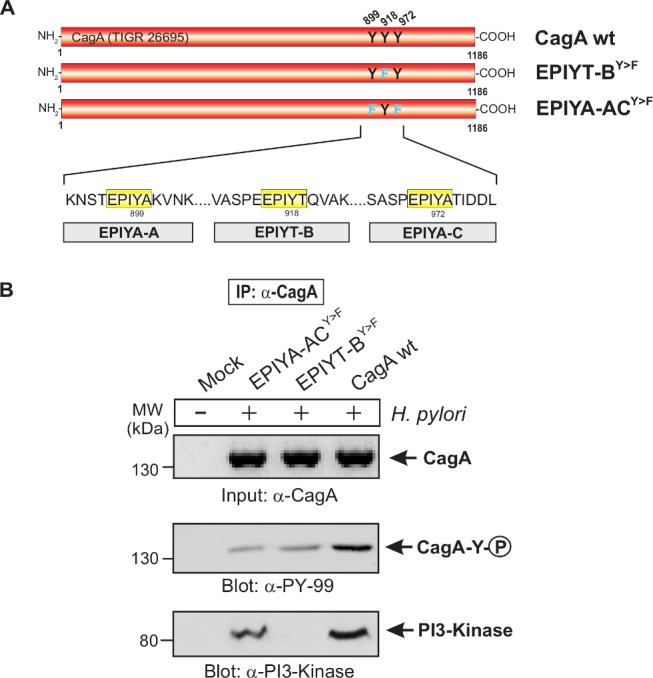
The EPIYT site at B-TPM of CagA is phosphorylated and necessary for interaction with PI3-kinase. **Panel A**: Site-directed mutagenesis of CagA TPM-motifs A, B and C was performed to generate the indicated phospho-resistant variants. Tyrosine residues in adjacent TPM-motifs were replaced by phenylalanines. The resulting single and double mutants are indicated and complemented into the *H. pylori* Δ*cagA* mutant. **Panel B**: AGS cells were co-cultured with the various CagA-expressing *H. pylori* strains for 6 h as indicated. Cell extracts were harvested and subjected to immunoprecipitation (IP) using α-CagA antibodies. CagA phosphorylation in the IPs was examined using α-pY-99 and α-CagA antibodies (arrows). All strains expressed similar amounts of CagA, and *H. pylori* expressing CagA wild-type (wt), EPIYT-AC^Y>F^, and EPIYT-B^Y>F^ all showed phosphorylation signal. Western blotting using α-PI3-kinase antibody revealed that only CagA wt and EPIYT-AC^Y>F^ can bind to PI3-kinase, but not the EPIYT-B^Y>F^ mutant, suggesting that EPIYT-B is phosphorylated and necessary for the interaction.

**Figure 5:**
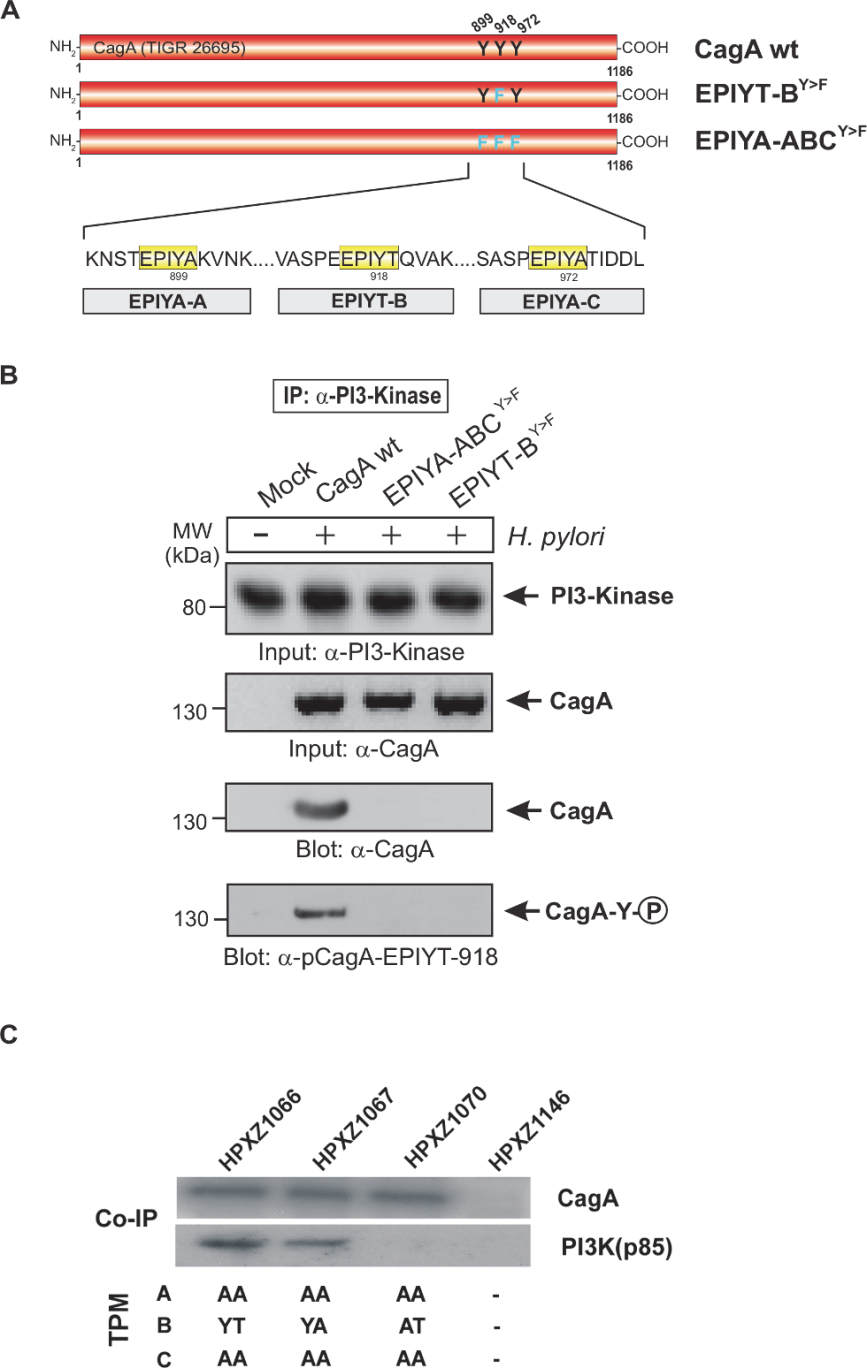
PI3-kinase can interact with B-TPM of CagA during co-culture. **Panel A**: Site-directed mutagenesis of CagA TPM-motifs A, B and C was performed to generate the indicated phospho-resistant variants. Tyrosine residues in adjacent TPM-motifs were replaced by phenylalanines. The resulting single and triple mutants were named as indicated and complemented into the *H. pylori* Δ*cagA* mutant. **Panel B**: AGS cells were co-cultured with the various CagA-expressing *H. pylori* strains for 6 h as indicated. Cell extracts were harvested and subjected to reverse immunoprecipitation (IP) using α-PI3-kinase antibodies. All samples contained similar amounts of PI3-kinase in the input control. CagA presence and phosphorylation at the EPIYT-site in the IPs was examined using phospho-specific α-pCagA-EPIYT-918 and α-CagA antibodies (arrows). Only the lane with *H. pylori* expressing CagA wt revealed a signal for CagA and phosphorylation at EPIYT-918 in the IP, indicating that phosphorylated EPIYT-B is necessary for the interaction with PI3-kinase. **Panel C**: After 24 h co-culture of AGS cells with the isogenic *H. pylori* strains containing the engineered CagA molecules, whole cell lysates were subjected to immunoprecipitation with an anti-CagA antibody. The anti-CagA immunoprecipitates (IP) were separated on SDS-PAGE, followed by western blot with anti-PI3-kinase (p85), which indicated that the engineered CagA B-EPIYA and CagA B-EPIYT molecules have different affinity to the PI3-kinase protein in AGS cells.

To investigate the interaction between the CagA B-TPM and PI3-kinase, we first used α-CagA antibodies to perform immunoprecipitation after co-culture of AGS cells with isogenic *H. pylori* 26695 strains with *cagA* variations. Western blotting using α-PI3-kinase antibody revealed that only wild-type CagA can bind to PI3-kinase but not EPIYA-ABC^Y>F^ or EPIYT-B^Y>F^ mutants (S3 Fig. in S1 Text). These data indicate that EPIYT B-TPM, but not EPIYA A- or EPIYA C-TPMs, is necessary for this interaction. The α-pY-99 control blot shows that the wild-type CagA is phosphorylated, while phosphorylated CagA with the EPIYT-B^Y>F^ mutation cannot interact with PI3-kinase (S3 Fig. in S1 Text). In a similar experiment, AGS cells were co-cultured with several isogenic CagA-expressing *H. pylori* strains including the EPIYT-AC^Y>F^ and EPIYT-B^Y>F^ mutants, followed by α-CagA immunoprecipitation. For both CagA variants (EPIYT-AC^Y>F^ and EPIYT-B^Y>F^), the phosphorylation signal was as expected. Western blotting using α-PI3-kinase antibody revealed that only CagA (wt) and EPIYT-AC^Y>F^ (with intact B-TPM) can bind to PI3-kinase, but not the EPIYT-B^Y>F^ mutant (Fig. 4). These findings indicate that EPIYT B-motif can be phosphorylated, which is necessary for the PI3-kinase-CagA B-TPM interaction. To further confirm our observation, CagA presence and phosphorylation at the EPIYT-site was examined using phospho-specific α-pCagA-EPIYT-918 and α-CagA antibodies when all samples contained similar amounts of PI3-kinase (Fig. 5A and B). Only the lane with *H. pylori* expressing wild-type CagA revealed a signal for CagA and phosphorylation at EPIYT B-TPM in the immunoprecipitation. These findings provide further evidence that phosphorylated EPIYT B-TPM is necessary for the interaction with PI3-kinase.

### The alternative B-TPM EPIYT has enhanced PI3-kinase affinity

Co-immunoprecipitation assays indicated that in AGS cells, CagAs possessing the EPIYA or EPIYT B-TPM interacted with PI3-kinase (p85), while CagA possessing EPIAT did not (Fig. 5C), suggesting that CagA activates PI3-kinase/AKT signaling pathways by interacting with the kinase via a functional B-motif. In AGS cells, CagA molecules possessing the B-domain EPIYT had higher affinity for PI3-kinase than those with EPIYA (Fig. 5C). These results suggest that the CagA interaction with PI3-kinase has activating, rather than inhibiting effects on its major downstream effector, AKT. The CagA molecules without a C-TPM sequence (as in CagA_147A_) did not induce AKT phosphorylation at T-308, indicating that the C-TPM sequence is necessary for expression of the B-domain function. The C-domain enhancement of B-domain activation of the PI3-kinase/AKT pathway is not dependent on the C-domain tyrosine phosphorylation since the B-domain EPIYT (in HPXZ1066) and B-domain EPIYA (in HPXZ1067) without C-domain activity activated the PI3-kinase/AKT pathway. This suggests the importance of maintaining the CagA dimerization state via the CagA multimerization (CM) sequence [39].

### Molecular modeling of CagA B-TPM interaction with the PI3-kinase SH2 domain

**Figure 6:**
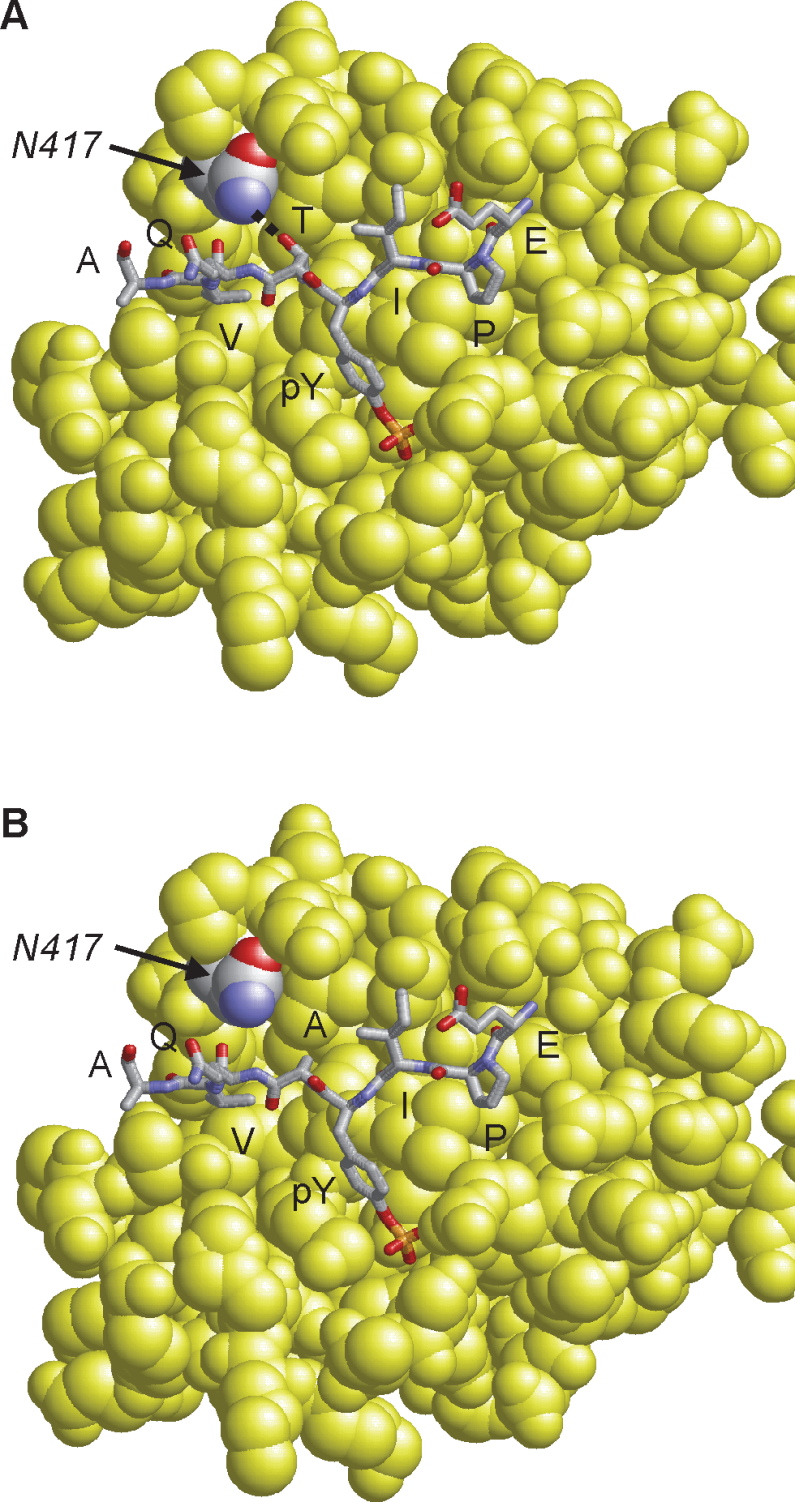
Model of the CagA B-TPM motif variants bound to PI3-kinase. The interactions of the EPIYTQVA motif (**Panel A**) are compared to that of the EPIYAQVA motif (**Panel B**). The threonine residue at the pY+1 position forms a side-chain hydrogen bond to N-417 of PI3-kinase, which cannot be formed by alanine. The motif is shown in stick presentation and colored according to atom type. The PI3-kinase SH2-domain is shown in yellow space-filled presentation and N-417 is colored by atom type.

Molecular modeling of the CagA B-TPM EPIYTQVA sequence in complex with the N-terminal PI3-kinase SH2-domain reveals that the threonine residue at the pY+1 position forms a side chain hydrogen bond with an asparagine residue (N-417) of PI3-kinase (Fig. 6A). The respective hydrogen bond cannot be formed for the “EPIYAQVA” motif, because the alanine present at the respective sequence position lacks the hydroxyl group side chain required for an interaction (Fig. 6B). Therefore, a T>A substitution at the pY+1 position is expected to significantly decrease binding affinity of the B-TPM motif to PI3-kinase. This model is also supported by experimental peptide binding studies, that show that threonine at the pY+1 position forms a stronger interaction than alanine with the respective SH2-domain [50]. Notably, PI3-kinase also contains a second SH2 domain, in which the asparagine required for ligand binding is conserved (N-707), suggesting that both PI3-kinase SH2-domains possess similar binding specificity for the pY+1 position.

### The alternative B-domain TPM EPIYT attenuates induction of the AGS cell hummingbird phenotype

**Figure 7:**
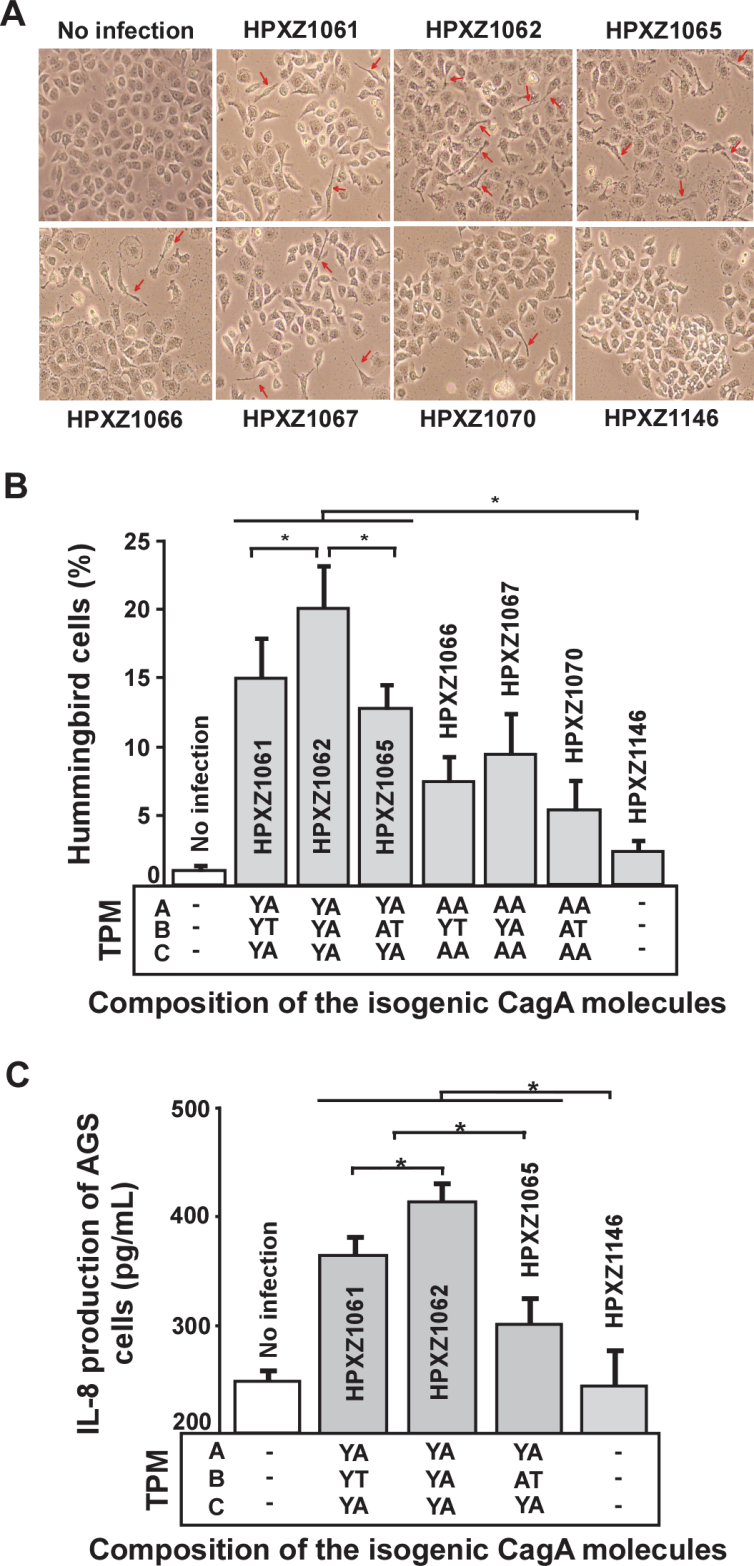
Analysis of the hummingbird phenotype and IL-8 induction after co-culture of human AGS cells with isogenic *H. pylori* strains containing the engineered CagA molecules. **Panel A**: AGS cells were co-cultured with the isogenic *H. pylori* strains at a multiplicity of infection (MOI) of 100:1 for 24 h; cell morphology was observed using microscopy. Red arrows indicate needle-like hummingbird cells. **Panel B**: The mean (+SD) proportion of hummingbird cells induced by the *H. pylori* isogenic *cagA* mutants during co-culture. *: p<0.05, Student’s T-test. **Panel C**: AGS cells were co-cultured with the isogenic *H. pylori* strains at a multiplicity of infection (MOI) of 100:1. The media were sampled at 24 h, centrifuged at 16,000 g, and supernatants collected. AGS cell IL-8 secretion (Mean+SD) was measured by an enzyme-linked immunosorbent assay. *: p<0.05, Student’s T-test.

CagA-positive *H. pylori* co-cultured with AGS cells induce an elongated cell morphology known as the hummingbird phenotype which is associated with effects on host cell polarity, migration, and adhesion [36,51]. Next we evaluated whether the major alternative B-domain TPM EPIYT affected CagA-induced hummingbird cell formation by co-culturing AGS cells with the isogenic *H. pylori* 26695 *cagA* mutants based on a comparable bacterial/host cell population level. We found that the isogenic *cagA+ H. pylori* strains induced significantly more hummingbird-type AGS cells than did an *H. pylori* Δ*cagA* mutant, but abolishing the A- and C- (EPIYA) TPMs significantly decreased hummingbird phenotype, indicating their involvement in hummingbird induction (Fig. 7A and B). In the presence of functional A and C TPMs, CagA with the B-domain EPIYA induced significantly more hummingbird cells than the CagA possessing the B-domain EPIYT or EPIAT (Fig. 7A and B). This observation suggests that the B-domain EPIYT has functional differences as compared to EPIYA. In the presence of non-functional (EPIAA) A- and C-TPMs, strains with CagA possessing EPIYT or EPIYA B-TPM induced significantly more hummingbird cells than the strain possessing the EPIAT B-TPM (p<0.05) (Fig. 7B). B-TPM functions may be affected by the A- and C-TPMs since the significant differential effects of EPIYT and EPIYA B-TPM were lost when we abolished those tyrosine phosphorylation sites.

### The alternative B-domain TPM EPIYT attenuates AGS IL-8 induction

Interleukin-8 (IL-8), a neutrophil-activating chemokine [52], may play an important role linking chronic inflammation and carcinogenesis [53]. The IL-8 induction effect is also associated with the number of C domains in Western CagA+ strains [49,54]. Here, we evaluated whether the major alternative B-domain TPM sequence, EPIYT, affects CagA-induced IL-8 production by co-culturing AGS cells with the isogenic *H. pylori* 26695 *cagA* mutants based on a comparable bacterial/host cell population level. At 24 h, AGS cells co-cultured with *H. pylori* Δ*cagA* mutants had the same IL-8 level as the AGS cells without *H. pylori* co-culture (control), but co-culture with the isogenic *H. pylori cagA* variants induced significantly higher IL-8 levels. Under these conditions, the isogenic *cagA* mutant containing the EPIYA B-TPM induced significantly more IL-8 induction than the mutant with the EPIYT B-TPM (Fig. 7C), a finding indicating differential EPIYA- and EPIYT-B TPM protein functions. The isogenic mutant with an EPIAT B-TPM had significantly decreased IL-8 induction (Fig. 7C). These results confirm that CagA can induce AGS cell IL-8 production via its B-domain TPM, and indicate the differential EPIYT and EPIYA functions.

## Discussion

A key host interaction factor of *H. pylori*, the CagA protein, has multiple polymorphisms which differ in their affinities to host interaction partners and in their regulation of gastric cell signaling cascades. EPIYA TPMs are critically important for CagA regulation of host signaling pathways [28,55], and the four types (A, B, C and D) have different host interaction partners [26] and/or varying affinities to the same partners [31,46], suggesting differential roles in regulation of host signaling pathways.

Matsunari *et al*. first reported there are three most common types of EPIYA sequences including the EPIYA, EPIYT and ESIYA, and that EPIYT of B-TPM is more predominant in Western CagAs [56]. In this study, we further reveal the A/T polymorphism that specifically occurs within the Western type B-domain, and we provide evidence for the first time that this polymorphism significantly affects CagA functions in host cells. Indeed, the B-domain polymorphisms of the Western strains differed in their correlation with upper GI tract diseases (Table 4), suggesting that a single SNP in a major bacterial interactive factor could decide disease outcome. The isogenic *H. pylori cagA* mutants expressing from the native genetic locus created for the present investigations may be valuable for further studies.

Through its B-domain, CagA interacts with host partners including the Shp2 phosphatase, Csk and PI3-kinases, as well as adaptor proteins Grb2 and Crk and the Shp1phosphatase, all of which carry SH2-domains [28]. Among these, Shp1 and Shp2 also interact with the A- or C-domains, Csk with the A-domain, Grb2 with C-domain, while PI3-kinase and CrkII only with the B-domain in a tyrosine-phosphorylation-dependent manner [26,28]. Different TPMs have both shared and specific host interaction partners suggesting that different EPIYA motifs could have specific roles in regulating host signaling pathways. Moreover, other motifs aside from the EPIYA TPMs could also be involved in CagA interactions with these host factors. CagA activation of PI3-kinase/AKT appears dependent on the CRPIA sequences [24], but activation of PI3-kinase/AKT also may be CagA-independent [37]. Our finding that *H. pylori* CagA with functional EPIYA or EPIYT B-domains binds with PI3-kinase confirms and extends the observations by Selbach *et al*. [28]. Recently, Lind *et al*. developed a novel strategy to systematically analyse phosphotyrosine antibodies recognizing single phosphorylated CagA EPIYA-motifs utilizing synthesized phospho- and non-phosphopeptides [57]. With this strategy, by generating and analyzing a novel phospho-specific CagA B-motif antibody (anti-pCagA-EPIYT-918) and isogenic CagA mutants with abolished TPMs, we further confirmed the CagA EPIYT-B domain tyrosine-phosphorylation status during *H. pylori* co-culture with host cells and revealed that tyrosine phosphorylation of the B-domain is necessary for the interaction between CagA and PI3-kinase. We observed that the EPIYT B-domain has higher affinity to PI3-kinase and greater AKT activation than the EPIYA B-domain. Our analysis by structural modeling of the CagA EPIYA and EPIYT B-motifs interacting with the SH2 domain of PI3-kinase further revealed the nature of differential interaction effects caused by the A/T polymorphism.

During gastric colonization, the host tyrosine kinases Src and Abl phosphorylate *H. pylori* CagA EPIYA motifs [21–23,25], which differ from the classical consensus phosphorylation sites in eukaryotic target factors (E-E-I-Y-E/G-X-F and I/V/L-Y-X-X-P/F of two tyrosine kinases, respectively) [58]. This suggests that the CagA EPIYA motif phosphorylation level in host cells may not be maximal. In that case, we propose that the A/T polymorphism/switch in the EPIYA motif could be important in regulation of the TPM phosphorylation efficiency and stability.

**Figure 8:**
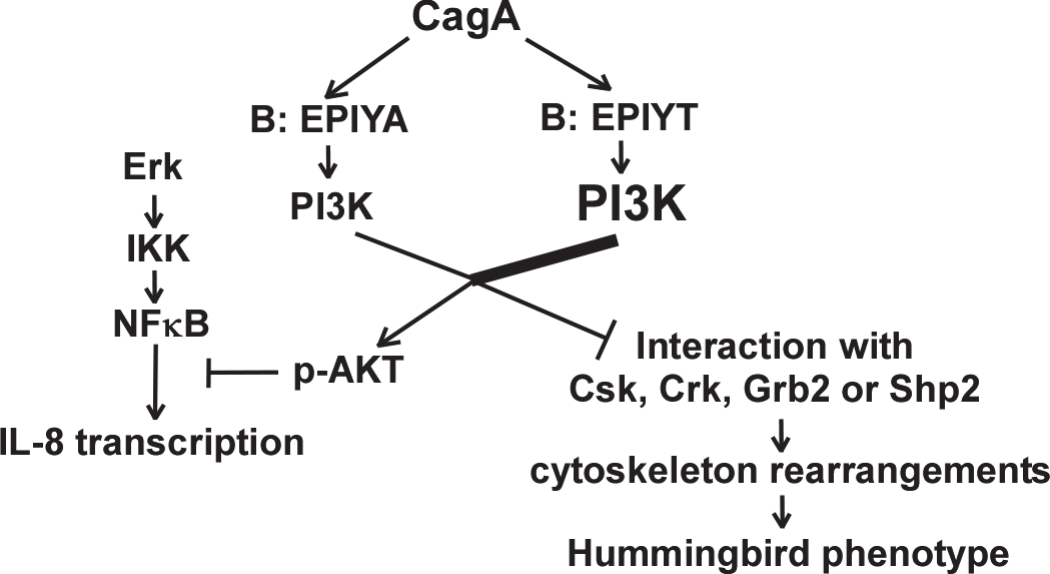
Proposed model showing that CagA may regulate activation of the PI3-kinase/AKT pathway through B-domain alternative TPM sequences. CagA proteins possessing a B-domain TPM with an EPIYT sequence may have more chance of tyrosine phosphorylation by host kinases. The different phosphorylation levels regulate CagA-tyrosine phosphorylation-dependent PI3-kinase interaction and AKT activation. Activated AKT further regulates AGS cell cytoskeleton rearrangements to affect CagA-induced hummingbird cell formation and inflammatory cytokine IL-8 secretion.

Strain-specific CagA sequence variation involves both conserved and non-conserved regions. CagA_147C_ used in our studies and CagA_26695_ used by Suzuki *et al*. share only 87.1% identify and have numerous SNPs flanking each EPIYA/T TPM as well as differing tagging. Considering the complex interactions between CagA with host protein partners as well as the complex signaling network, use of transfected protein-expressing systems and both technical and structural differences may affect signaling. The number of *H. pylori* CagA molecules within host cells in different assays (e.g. CagA transfection and co-culture with *H. pylori* with native expressing CagA) could markedly vary with differing kinetics of CagA phosphorylation, leading to different outcomes. Increased AKT activation and decreased IL-8 secretion of AGS cells [59], and PI3-kinase/AKT pathway repression of IL-8 production during *Salmonella* co-culture with intestinal epithelial cells [60] have been described. Strain- and time-dependent *H. pylori* CagA-mediated IL-8 induction in AGS cells occurs through the Erk and NF-κB pathways [49,61–64]. CagA-mediated PI3-kinase/AKT activation attenuates IL-8 induction by repressing Erk/NF-κB including through the Shp-2/Erk pathway [49], the Shp2-independent Ras/Raf/Mek/Erk pathway [65], and by Ras-independent Erk activation [66]. Inactivating B-TPM abolished PI3-kinase/AKT activation, but decreased IL-8 secretion. Through B-TPM, CagA also may interact with multiple other proteins in a site-competition and/or time-dependent manner. For example, CagA TPMs interact with Shp2 through phosphorylated EPIYAs [28,34] enhancing Shp2 activity and Erk phosphorylation [31]. The B-TPM could positively regulate IL-8 production through activating the Shp2/Erk/ NF-κB pathway [49]. Abolishing the isogenic single B-TPM inactivated the IL-8-repressing PI3-kinase/AKT, but also inactivated IL-8-stimulating Shp2/Erk. Repression of IL-8 production by the B-TPM-mediated PI3-kinase/AKT effect reflects cross-talk between the PI3-kinase/AKT and Shp2/Erk pathways. Consistent with the overall differential signaling, Western *cagA+ H. pylori* strains with EPIYT or EPIYA B-TPMs are associated with different patterns of clinical outcomes (Table 4 and Fig. 8), an observation that needs to be confirmed. The clinical outcome of *H. pylori* colonization results from long-term processes, and therefore, how the B-TPM-mediated PI3-kinase/AKT effect alters host gastric cancer development in long-term *H. pylori* colonization deserves further investigation. The PI3-kinase/AKT signaling pathway controls many of the hallmarks of cancer, and many tumor tissues have enhanced PI3-kinase/AKT activities [67,68]. However, PI3-kinase/AKT and their effectors are pleiotropic and have complex crosstalk and feedback behaviors in the signaling network, which are not fully known [67,68]. We studied the regulation of CagA on PI3-kinase/AKT pathway *in vitro* at an early time of bacterial interaction with host cells, while long-term studies in mice or observations in patients at risk for gastric cancer will help resolving the clinical significance of the polymorphism.

*H. pylori* colonization induces AGS cell scattering and elongation (hummingbird phenotype) through multiple CagA-related mechanisms; CagA binds to Csk through its A- or B-TPMs, inhibiting SFK activity, and binds to Shp2 through its A-, B- or C-TPMs, leading to FAK dephosphorylation [35,69]. Attenuated hummingbird phenotype induction present in the *cagA* mutant with the EPIYT B-TPM (vs. EPIYA) reflects differential B-domain functional roles, possibly through modulating direct interactions with Csk or Shp2. Inhibition of AKT activation by the PI3-kinase inhibitor LY294002 has no effect on the hummingbird phenotype [36]. However, LY294002 inhibits PI3-kinase catalysis by competing for ATP binding [70], but does not directly affect the p85 binding activity with tyrosine-phosphorylated motifs such as the CagA B-TPM. Such findings suggest that hummingbird induction by the CagA B-TPM relates to the competition between PI3-kinase and Csk or Shp2 for binding at the B-TPM, but is not directly related to PI3-kinase activity.

The A- and B-domains have unique host interacting partners, such as Csk, which do not interact with C- or D-TPMs. These domains could possibly attenuate the C-domain-Shp2-interaction by binding with Csk to inactivate SFK members [71]. In this model, the C- and D-TPMs serve as the primary phosphorylation motifs interacting with host signaling partners, and the A- and B-TPMs serve as secondary sites, phosphorylated after C- or D-TPMs [25], suggesting a potential regulatory role through competition between different TPMs. The EPIYT/EPIYA B-TPM polymorphism that we studied provides a new level of complexity in *H. pylori* colonization and pathophysiology.

## Materials and Methods

### Bacterial strains, media and growth conditions

*H. pylori* strain 26695 was used to construct a series of isogenic *cagA* mutants, which express CagA variants from the native CagA genetic locus [72]. *H. pylori* strains 147C and 147A, a pair of naturally occurring isogenic *cagA* strains with EPIYA ABC and AB motifs, respectively [49], were used as isogenic *cagA* sequence templates. *H. pylori* CagA-expressing strains P227, Oki-61, P341, 2002-370 and 26695, also were used for AGS co-culture and evaluation of CagA phosphorylation [73]. The *H. pylori* strains were grown at 37°C in 5% CO_2_ on trypticase soy agar (TSA) plates with 5% sheep blood (TSA, BBL Microbiology Systems, Cockeysville MD) or Brucella agar plates (BA, Difco Laboratories, Detroit MI) supplemented with 10% newborn calf serum (NBCS; Serologicals Corporation, Norcross GA) and suitable antibiotics [74]. Antibiotic-resistant isogenic *H. pylori* strains were selected with kanamycin (Km; 10 μg/mL) or chloramphenicol (Cm; 30 μg/mL), as appropriate. Alternatively, *H. pylori* strains were grown in thin layers on horse serum GC agar plates supplemented with vancomycin (10 μg/mL), nystatin (1 μg/mL), and trimethoprim (5 μg/mL), and for defined mutants with Cm (6 μg/mL) and/or Km (8 μg/mL) at 37°C for 2 days in an anaerobic jar containing a Campygen gas mixture of 5% O2, 10% CO2, and 85% N2 (Oxoid, Wesel, Germany) [75]. *E. coli* DH5α was grown in Luria-Bertani (LB) medium at 37°C [76]. Ampicillin (Ap; 100 μg/mL), Cm (30 μg/mL) or Km (50 μg/mL) were used for selecting vectors or the constructs in *E. coli* during cloning.

### Construction of isogenic *H. pylori* strains

Western type *H. pylori* strain 147C *cagA* has an EPIYT B-TPM, as well as one EPIYA -A and -C TPM (*cagA*_147C B:EPIYT A&C:Y_) [49]. To evaluate B-TPM A/T polymorphism effects on CagA functions, the threonine site of EPIYT B-TPM was replaced with alanine by recombination-PCR mediated site-directed mutagenesis, leading to the generation of the isogenic *cagA, cagA*_147C B:EPIYA A&C:Y_. To evaluate B-TPM A/T polymorphism effects on tyrosine phosphorylation of CagA B-TPM, the two tyrosine phosphorylation sites, A- and C-TPMs of the wild-type *cagA* (*cagA*_147C B:EPIYT A&C:Y_) and the isogenic *cagA* (*cagA*_147C B:EPIYA A&C:Y_), were replaced with alanine, leaving the B-TPM as the only functional tyrosine phosphorylation site. This resulted in two isogenic *cagA*s: *cagA*_147C B:EPIYT A&C:Y>A_ and *cagA*_147C B:EPIYA A&C:Y>A_. For controls, the tyrosine of B-TPMs of the wild-type and the isogenic *cagAs* were further replaced with alanine to abolish B-TPM tyrosine phosphorylation function, leading to *cagA*_147C B:EPIAT A&C:Y_ and *cagA*_147C B:EPIAT A&C:Y>A_. Each of the wild-type and isogenic *cagA* genes was first fused at the 3’ end to the hemagglutinin (HA) tag and then linked to a 617 bp *cagA* downstream region sequence based on the 26695 genomic sequence [72]. An *aphA* (Km^R^) cassette [77] was inserted between the *cagA*-HA fusion gene and the *cagA* downstream region sequence as a selection marker for the *H. pylori* mutant construction. Each construction was cloned into the vector pGEM-T easy (Promega, Madison WI), creating plasmids pXZ476, pXZ465, pXZ468, pXZ471, pXZ472, and pXZ475, which carry *cagA*_147C B:EPIYT A&C:Y_, *cagA*_147C B:EPIYA A&C:Y_, *cagA*_147C B:EPIAT A&C:Y_, *cagA*_147C B:EPIYT A&C:Y>A_, *cagA*_147C B:EPIYA A&C:Y>A_, and *cagA*_147C B:EPIAT A&C:Y>A_, respectively (S2 Table in S1 Text). To replace the *cagA*_26695_ sequence of *H. pylori* strain 26695 with the isogenic *cagA*_147C_ sequences, a truncated *cagA*_147CN_ (2445 bp) lacking C-terminal A- B- or C-TPMs was cloned and linked with a *cat* (Cm^R^) cassette [77] and then with the 617 bp *cagA* downstream region sequence based on the 26695 sequence using pGEM-T easy, creating pXZ478 (S2 Table in S1 Text). To construct the *H. pylori* Δ*cagA* control, the *cagA* upstream (839 bp) and downstream (1076) region sequences of *H. pylori* 26695 were linked and inserted with an intervening *sacB-cat* (Cm^R^) cassette to replace the entire *cagA_26695_* ORF on the same vector, creating plasmid pXZ083 (S2 Table in S1 Text).

To express the series of mutant *cagA* genes from the *cagA* native genetic locus in the 26695 genetic background, we first replaced the *cagA*_26695_ sequence on the 26695genome with a *cagA*_147C_s via homologous recombination (S1 Fig. in S1 Text). The wild-type *H. pylori* strain 26695 was transformed by plasmid pXZ478 to Cm^R^ to create strain HPXZ1043 with isogenic *cagA*_147CN_ replacing native *cagA*_26695_. DNA sequencing confirmed the replacement of *cagA*_26695_ with the *cagA*_147CN_ sequence in the 26695-derived Cm^R^/Km^S^ strain HPXZ1043, and western blot confirmed the expression of the truncated CagA_147CN_ protein from the native locus and the CagA promoter. Strain HPXZ1043 Cm^R^/Km^S^ was then transformed to Km^R^/Cm^S^ with plasmids, pXZ476, pXZ465, pXZ468, pXZ471, pXZ472, or pXZ475, to create mutants HPXZ1061 (*cagA*_147C B:EPIYT A&C:Y_), HPXZ1062 (*cagA*_147C B:EPIYA A&C:Y_), HPXZ1065 (*cagA*_147C B:EPIAT A&C:Y_), HPXZ1066 (*cagA*_147C B:EPIYT A&C:Y>A_), HPXZ1067 (*cagA*_147C B:EPIYA A&C:Y>A_) and HPXZ1070 (*cagA*_147C B:EPIAT A&C:Y>A_), respectively. The wild-type *H. pylori* strain 26695 was transformed to Cm^R^ with pXZ083 to create the *cagA*-negative mutant HPXZ1146 (Δ*cagA*::*sacB-cat*) (S2 Table in S1 Text). To confirm each construction, sequencing of related regions was performed at Macrogen (Rockville MD), and all sequence analysis was performed using Sequencher (Gene Codes, Ann Arbor MI).

### Cloning, complementation and site-directed mutagenesis of CagA using shuttle vector pHel3

To analyse the EPIYA- and EPIYT-motifs in the CagA protein, the complete *cagA* gene of *H. pylori* strain 26695 (accession number: AAD07614) containing its promoter was amplified by PCR, cloned into the pCR2.1 vector (Invitrogen) and sequenced [73]. For construction of a complementation vector, this *cagA* fragment was cloned in the *E. coli/H. pylori* shuttle vector pHel3 containing the *oriT* of RP4 and a kanamycin resistance gene cassette (Aph-A3) as a selectable marker, resulting in vector pSB19 [73]. Site-directed mutagenesis of tyrosines Y-899, Y-918 and Y-972 in the CagA sequence was done using the Sculptor mutagenesis kit, as directed (Amersham Pharmacia Biotech) and resulting plasmids were transformed into *H. pylori* isogenic Δ*cagA* mutant [78], as described [79].

### Synthesis of phospho- and non-phospho CagA TPM peptides

The C-STEPIYAKVNK (EPIYA-A), C-STEPI(pY)AKVNK (phospho-EPIYA-A), C-PEEPIYTQVAK (EPIYT-B), C-PEEPI(pY)TQVAK (phospho-EPIYT-B), C-PEEPIYAQVAK (EPIYA-B), C-PEEPI(pY)AQVAK (phospho-EPIYA-B), C-SPEPIYATIDD (EPIYA-C) and C-SPEPI(pY)ATIDD (phospho-EPIYA-C) amino acid sequences were synthesized by Jerini AG (Berlin, Germany). These 11-mer peptides were chosen because prior studies have shown that α-phosphotyrosine antibodies typically recognize short phosphopeptides, and 11-mer and 9-mer sequences are both necessary and sufficient [57]. Commonly, 11-mer peptides also are used for immunizations to generate phospho-specific antibodies, which then recognize the corresponding phosphopeptides bound to affinity columns and in ELISA (Biogenes, Berlin, Germany). All above EPIYA and EPIYT peptides were purified by HPLC, and full-length synthesis as well as purity of each peptide was confirmed by mass spectrometry by Jerini AG. The peptides were resolved at a concentration of 1 mg/mL in DMSO and stored at −20°C.

### Dotblot analysis

Twenty μg of each CagA peptide were mixed in 1 mL of TBST blotting buffer (140 mM NaCl; 25 mM Tris-HCl, pH 7.4; 0.1% Tween-20). These peptide samples were spotted onto Immobilon-P membrane (Merck Millipore, Darmstadt, Germany) using the BioDot SF apparatus (Bio-Rad, Munich, Germany). The resulting Dotblots were dried and subjected to antibody detection as described below for Western blots [57].

### AGS culture and co-culture assays

Human gastric epithelial (AGS; ATCC CRL 1739) cells (obtained from American Type Culture Collection) were cultured at 37°C in a humidified atmosphere with 5% CO2 in RPMI 1640 (Invitrogen, Carlsbad CA) with 10% fetal bovine serum (FBS; Invitrogen) with antibiotic-antimycotic mixture (1X; Life Technologies, Grand Island NY) [80]. Before co-culture experiments, AGS cells (2×10^5^cells/well) were transferred to a new 6-well plate and incubated in fresh RPMI 1640 with 10% FBS and antibiotic-antimycotic for 24 h. The attached AGS cells were washed and incubated in the RPMI 1640 media without serum or antibiotic for 16 h. The AGS cells were then co-cultured with PBS-prewashed *H. pylori* cells, which were collected from 24-h TSA plates at a multiplicity of infection (MOI) of 100:1 and from fresh serum- and antibiotic-free RPMI 1640 for 8–24 h.

### Cell elongation assays

AGS cultures and the co-culture of AGS and *H. pylori* were grown on coverglass (Fisher Scientific, Pittsburgh PA) in 6-well plates [81]. After 24-h co-culture, 10 random fields of view for each coverglass were examined at a magnification of ×200 using a Leica DMI 6000B microscope. Alternatively, the AGS cells were co-cultured with *H. pylori* cells at MOI of 50 for 6 h, when the cells were harvested in ice-cold PBS containing 1 mmol/L Na_3_VO_4_ (Sigma-Aldrich). Elongated AGS cells in each experiment were quantitated in 10 different 0.25-mm^2^ fields using an Olympus IX50 phase contrast microscope [82]. All experiments were performed in triplicate.

### IL-8 assay

After 24-h incubation, co-culture media were sampled and centrifuged at 16,000 g, and supernatants collected. AGS cell IL-8 secretion was measured by an enzyme-linked immunosorbent assay using the Human IL-8 ELISA Kit II (BD Biosciences, San Jose CA), in accordance with the manufacturer’s instructions.

### Generation of phospho-specific and non-phospho-specific α-EPIYT antibodies

Phospho-specific and non-phospho polyclonal rabbit CagA antibodies were raised against peptides corresponding to the following amino acid residues derived from the B-TPM motif of strain 26695: C-PEEPIYTQVAK (non-phospho-EPIYT-B) and C-PEEPI(pY)TQVAK (phospho-EPIYT-B). For this purpose, both peptides were conjugated to *Limulus polyphemus* haemocyanin carrier protein and two rabbits each were immunized by Biogenes GmbH (Berlin, Germany), according to standard protocols. The resulting phospho-specific antibodies (α-pCagA-EPIYT-918) were affinity-purified against the corresponding non-phospho peptide bound to a column. The resulting non-phospho antibodies (α-CagA-EPIYT-918) were affinity-purified against the corresponding phospho-peptide. Both antibodies were prepared and purified by Biogenes GmbH (Berlin, Germany). Their specificity was confirmed by dot blotting against the phospho- and non-phospho peptides (S2 Fig. in S1 Text).

### Immunoblotting, immunoprecipitation, and antibodies

To prepare whole cell extracts for immunoblotting, media were removed after 24-h incubation and AGS cells were washed with ice-cold PBS 5 times to remove *H. pylori* cells. The whole cell lysates for western blotting were prepared with RIPA lysis buffer (Thermo Scientific Pierce, Rockford IL) with Halt Protease and Phosphatase Inhibitor Cocktail (Thermo Scientific Pierce). Lysates were separated by SDS-PAGE (Expedeon Inc. San Diego CA) and transferred to Immobolin-P PVDF Transer Membrane (Fisher Scientific). Membranes were blocked in TBST with 3% BSA or 5% skim milk for 1 or 2 h at room temperature. Membranes were incubated with the following antibodies according to the instructions of the manufacturer. Immunodetection of CagA peptides and the various proteins of interest were performed using horseradish peroxidase–conjugated anti-mouse or anti-rabbit polyvalent sheep immunoglobulin secondary antibodies and using chemiluminescence reagents, West Femto Chemiluminescent Substrate (Thermo Scientific Pierce) or Amersham ECL Western Blotting Detection Reagents (GE Healthcare, Piscataway NJ) in accordance with the manufacturers’ instructions [83]. After exposure to X-ray film, the target band intensities were quantified using ImageJ software (NIH, Bethesda MD). For immunoprecipitation, the whole cell lysates were prepared with IP Lysis/Wash buffer (Thermo Scientific Pierce, Rockford IL) with Halt Protease and Phosphatase Inhibitor Cocktail (Thermo Scientific Pierce). Immune complexes were prepared using Pierce Crosslink Immunoprecipitation kit (Thermo Scientific Pierce) in accordance with the manufacturers’ instructions. The immunoprecipitates were subjected to SDS-PAGE, as described above. Anti-actin, anti-AKT, anti-phospho-AKT (pThr308), and anti-phospho-AKT (pSer473) ePI3-kinase were obtained from Cell Signaling Technology (Danvers MA). Anti-HA, anti-Shp2, anti-Crk II, and anti-phospho-CrkII (pTyr221) were obtained from Thermo Scientific Pierce, anti-phosphotyrosine was obtained from EMD Millipore Inc. (Billerica MA), anti-Csk, anti-phospho-Csk (pSer364), anti-GAPDH and pan-phosphotyrosine antibody pY-99 were obtained from Santa Cruz Biotechnology, Inc. (Santa Cruz CA), and anti-CagA was produced as described [49]. Anti-PI3-kinase (p85) antibodies were obtained from Cell Signaling Technology (Danvers MA) or Santa Cruz Biotechnology, Inc. (Santa Cruz CA). Rabbit polyclonal and mouse monoclonal α-CagA antibodies were from Austral Biologicals, or from Emd Millipore Corporation (Billerica MA).

The phosphorylation status of CagA and bound PI3-kinase also was verified by immunoprecipitation experiments as described [73,79]. Briefly, co-cultured or control AGS cells were washed with cold PBS and lysed for 30 min at 4°C in lysis buffer (20 mM Tris pH 7.2, 150 mM NaCl, 5 mM EDTA, 1% Triton X-100, 10% glycerol, 1 mM Na3VO4, COMPLETE™ inhibitor mix from Roche). Lysates were pre-cleared with protein G-Sepharose (Pharmacia, Uppsala, Sweden) for 2 h at 4°C. Two micrograms of the monoclonal α-CagA antibody (Austral Biologicals, San Ramon CA) or polyclonal antibody against the p85 subunit of PI3-kinase (Santa Cruz Biotechnology, Dallas TX) were added to the supernatant and incubated overnight at 4°C on a shaker. Immune complexes were precipitated by addition of protein G-sepharose for 2 h, washed three times in 0.5× PBS and then mixed with equal amounts of 2× SDS-PAGE buffer. Precipitates were analyzed by SDS-PAGE and immunoblotting.

### Quantification of Dotblot and Western blot signals

Spot or band intensities on blots probed with the different α-phosphotyrosine antibodies were quantitated with the Lumi-Imager F1 (Roche Diagnostics, Mannheim, Germany). Densitometric measurement of signal intensities revealed the percentage of phosphorylation per sample [84].

### 3D-Modeling

The CagA-PI3-kinase interaction was modeled using the crystal structure of the N-terminal PI3-kinase SH2-domain in complex with a C-kit phosphotyrosyl peptide (PDB: 2IUH) as a template [85]. Modeling of the Cag-A B-TPM motif was performed using SwissModel [86] and included the sequence stretches “EPIYTQVA” or “EPIYAQVA”. Structural analysis and visualization was performed using RasMol [87].

### Assessment of CagA EPIYA motif polymorphisms

A total of 2561 *H. pylori* complete or partial CagA protein sequences available at GenBank on August 8^th^ 2013 were collected. The *cagA* EPIYA A-, B-, C-, and D-TPM types were defined as described [46]. The numbers of each type of EPIYA TPMs and the polymorphisms within the five specified amino acids were tabulated independently three times. A Chi-square test was performed to evaluate the variance in the representation of each EPIYA TPM.

## Supporting Information

S1 Text The supporting Information file includes S1, S2, and S3 Fig., and S1 and S2 Tables. (DOCX)
