# Supplementary material for: A Specific A/T Polymorphism in Western Tyrosine Phosphorylation B-Motifs Regulates Helicobacter pylori CagA Epithelial Cell Interactions
Source: PLoS Pathog. 2015 Feb 3;11(2):e1004621. doi: 10.1371/journal.ppat.1004621 (PMC4412286; doi:10.1371/journal.ppat.1004621)
Supplement: S1 Text — (DOCX) [file ppat.1004621.s001.docx]

**Supporting Information**

**Supplemental Figure Legends**

**Supplemental Figure S1. Construction of *H. pylori* isogenic *cagA* mutants with TPM polymorphisms. Panel A:** Schematic presentation of the seven constructs used for epithelial cell co-culture studies. **Panel B:** Expression of the isogenic *cagA* variants from each constructed *H. pylori* mutant was confirmed using whole-cell lysates and Western blot analysis with anti-CagA antibody. The colors for TPMs are: EPIYA (red), EPIYT (green), EPIAA (black), and EPIAT (gray).

**Supplemental Figure S2.** **Generation and analysis of** **phospho-specific and non-phospho antibodies against the EPIYT B-TPM motif of *H. pylori* CagA. Panel A:** Many CagA proteins of *H. pylori* (e.g. strain 26695) contain the EPIYA-A, EPIYT-B, and EPIYA-C segments as indicated. These sites represent the tyrosine phosphorylation motifs (TPMs), which can be phosphorylated by c-Abl and c-Src host kinases. **Panel B:** The indicated 11-mer phospho- and non-phospho peptides of each TPM site were synthesized and immobilized on PVDF membranes using a Dotblot apparatus. All Dotblots were probed with α-pCagA-EPIYT-918 (phospho) or α-CagA-EPIYT-918 (non-phospho) antibodies and exposed as described in the Materials & Methods section. The results show that 11-mer peptides of B-TPM are sufficient for strong and specific recognition by the antibodies. The blot with the commercial pan-phosphotyrosine antibody α-PY99 served as loading control for all three phospho-peptides. **Panel C:** Phospho- and non-phospho peptides of the B-TPM motif were generated carrying a T>A mutation at amino acid position 919, resulting in the conventional EPIYA-B sequence. The Dotblots show that the mutated phospho-peptide also can be detected by this method using the α-pCagA-EPIYT-918 (phospho) antibody, while the corresponding non-phospho peptide cannot.

**Supplemental Figure S3. The EPIYT site at the CagA B-TPM is necessary for interaction with PI3-kinase. Panel A:** Site-directed mutagenesis of CagA TPM-motifs A, B and C was performed to generate the indicated phospho-resistant variants. Tyrosine residues in adjacent TPM-motifs were replaced by phenylalanines. The resulting triple and single mutants are indicated, and were complemented into *H. pylori* Δ*cagA* mutant. **Panel B:** AGS cells were co-cultured with the various CagA-expressing *H. pylori* strains for 6 h as indicated. Cell extracts were harvested and subjected to immunoprecipitation (IP) using α-CagA antibodies. CagA phosphorylation in the IPs was examined using α-pY-99 and α-CagA antibodies (arrows). All strains expressed similar amounts of CagA, but only *H. pylori* expressing wild-type (wt) CagA and EPIYT-B^Y>F^ revealed a phosphorylation signal. Western blotting using α-PI3-kinase antibody revealed that only CagA wt can bind to PI3-kinase but not EPIYA-ABC^Y>F^ and EPIYT-B^Y>F^ mutants, suggesting that EPIYT-B but not EPIYA-A or EPIYA-C is necessary for the interaction.

**Supplemental Table Legends**

**Supplemental Table S1. Single base polymorphisms at the YA or YT position of 1021 Western CagA B-TPM.**

**Supplemental Table S2. Construction of *H. pylori* strains with isogenic *cagA* TPMs**

**Supplemental Table S1. Single base polymorphisms at the YA or YT position of 1021 Western CagA B-TPM.**

|  |  | | **B-TPM** | | | **Other *H. pylori* loci for 26695^a^** | | |  |
| --- | --- | --- | --- | --- | --- | --- | --- | --- | --- |
| **Amino**  **acid** | | **Codon** | | **Number**  **analyzed** | **Number** | | **Number**  **analyzed** | **Number** | **p-value^b^** |
| **YT** | | TAT ACT | |  | 370 | |  | 27 |  |
|  |  | TAC ACT | | 407 | 36 | | 146 | 83 | <0.001 |
|  |  | TAC ACA | |  | 1 | |  | 36 |  |
| **YA** | | TAC GCT | |  | 365 | |  | 182 |  |
|  |  | TAT GCT | | 614 | 248 | | 363 | 102 | <0.001 |
|  |  | TAC GCA | |  | 1 | |  | 79 |  |

^a^ Source.

^b^ Chi square analysis.**Supplemental Table S2. Construction of *H. pylori* strains with isogenic *cagA* TPMs^a^.**

|  | **Amino acid sequence** | | | **Domains with**  **intact tyrosine**  **phosphorylation sites** |
| --- | --- | --- | --- | --- |
| **HP strain** | **A-TPM (EPIYA)^b^** | **B-TPM** | **C-TPM (EPIYA)^b^** |  |
| HPXZ1061 | YA | EPIYT | YA | A, B, C |
| HPXZ1062 | YA | EPIYA | YA | A, B, C |
| HPXZ1065 | YA | EPIAT | YA | A, C |
| HPXZ1066 | AA | EPIYT | AA | B |
| HPXZ1067 | AA | EPIYA | AA | B |
| HPXZ1070 | AA | EPIAT | AA | - |

^a^ All have a C-terminal HA tag.

^b^ Wild-type is EPIYA; the engineered mutants all are EPIAA.

**
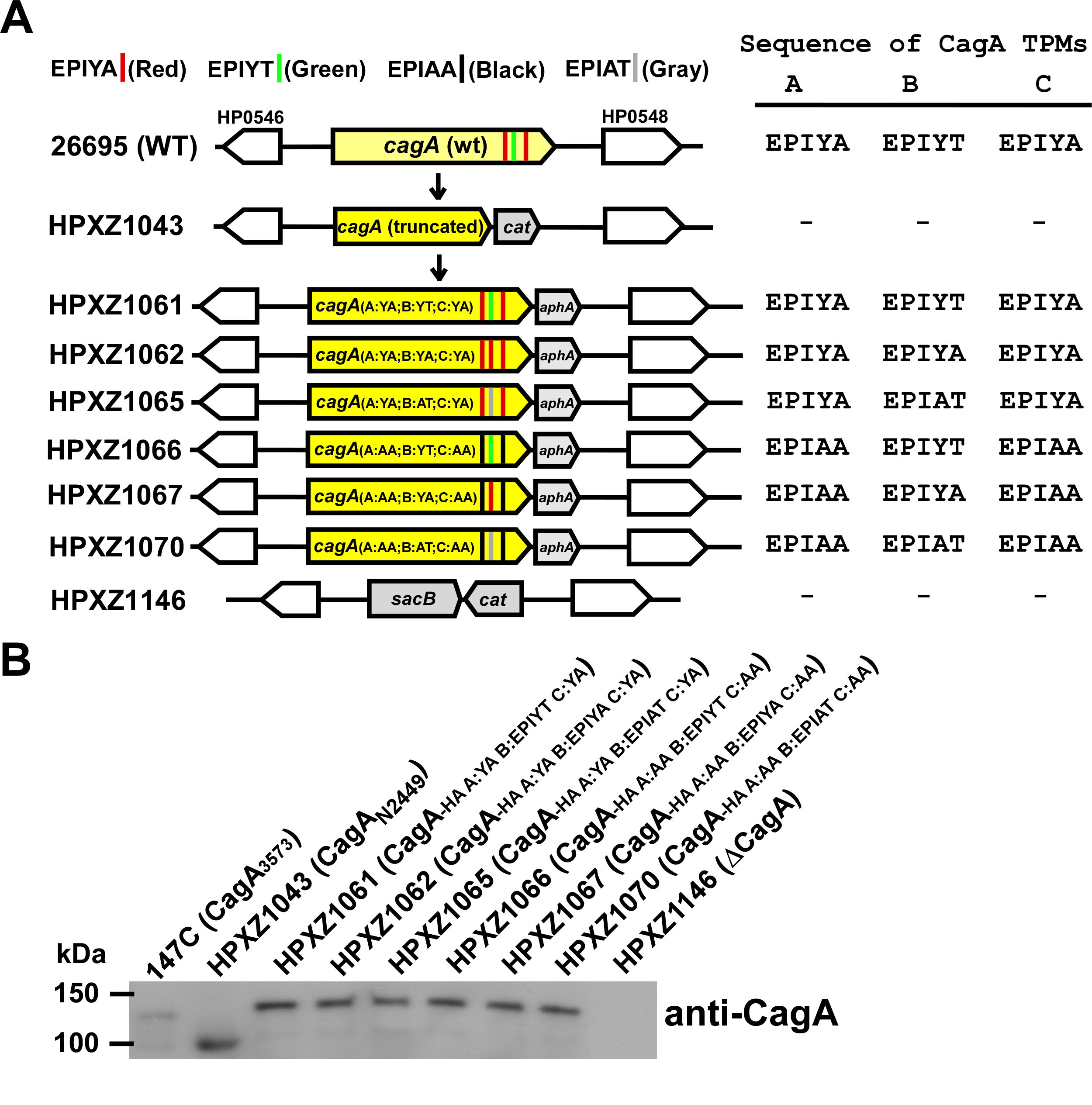
**

**Supplemental Figure S1.**

**
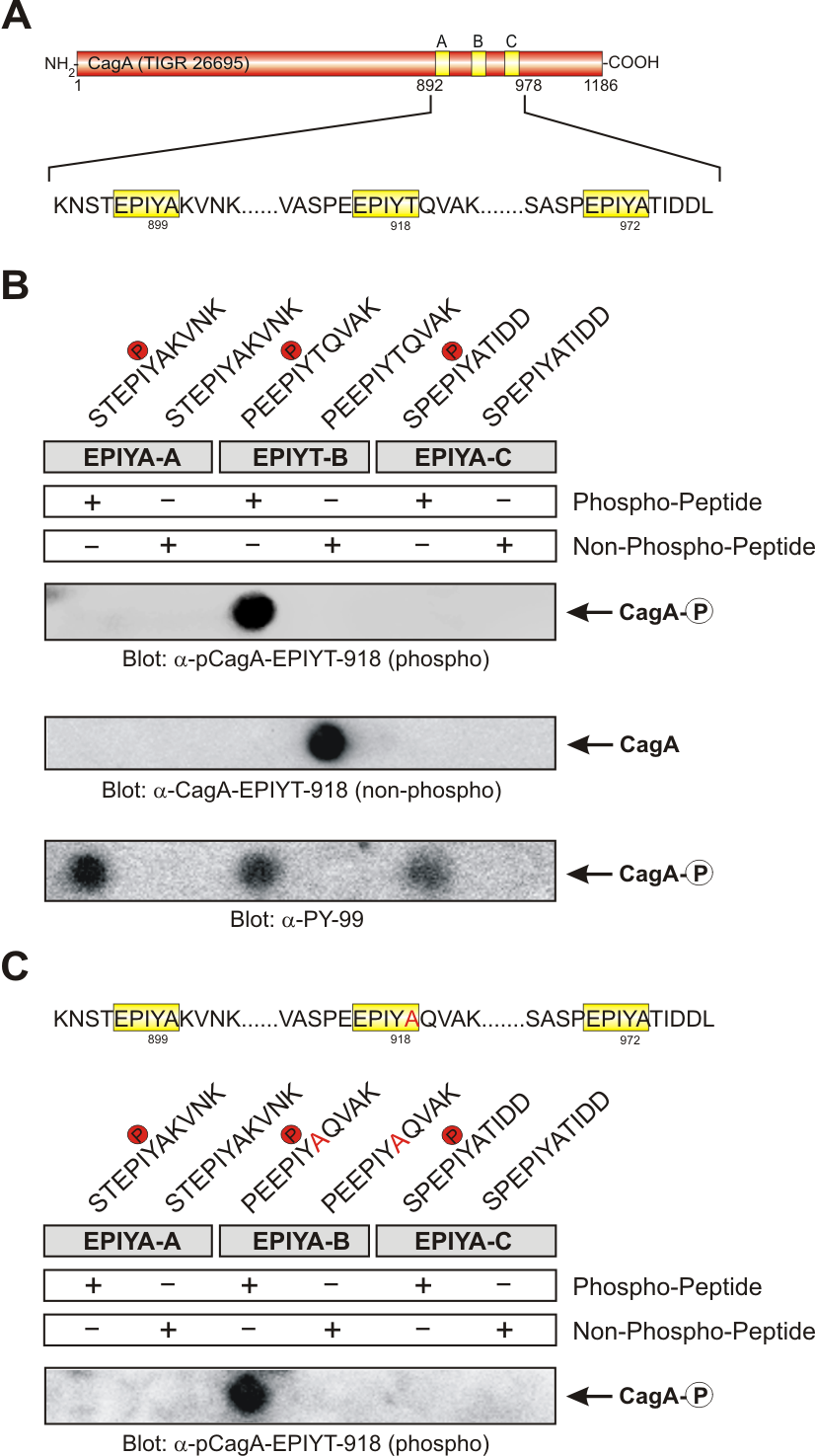
**

**Supplemental Figure S2.**

**
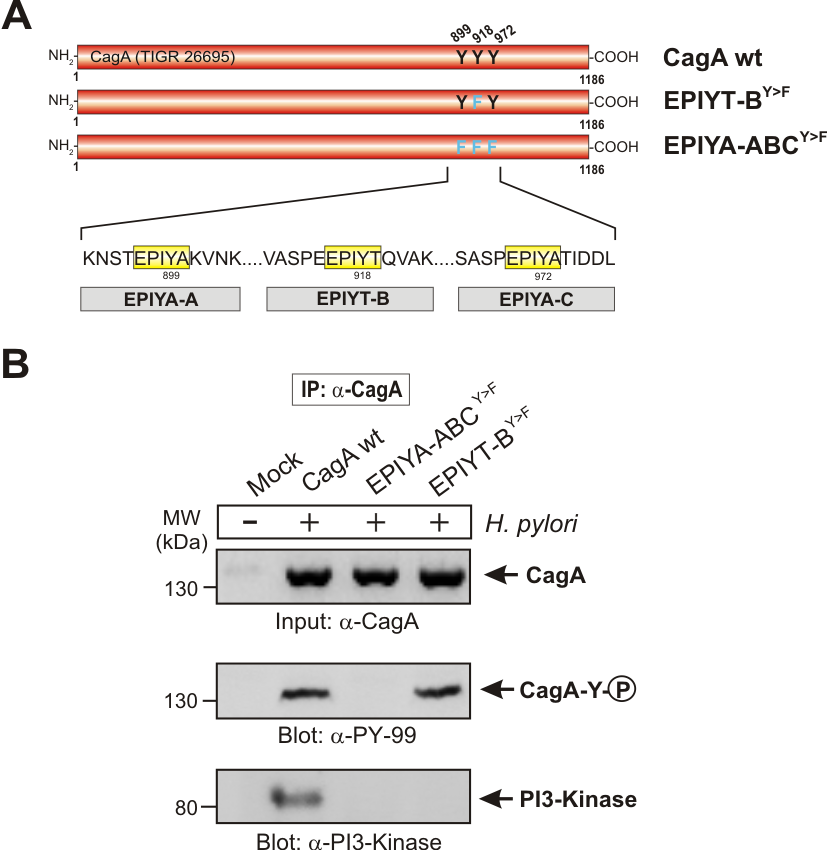
**

**Supplemental Figure S3.**
